# Old Europe — New Public Health

**Published:** 2007-06-15

**Authors:** Ilona Kickbusch

**Affiliations:** Graduate Institute of International Studies

In his wonderful book *The Great Influenza*, John M. Barry describes how the distinguished innovators of American medical education and academic public health traveled to Europe at the end of the 19th century to glean all they could for creating a more advanced model for U.S. schools of medicine (1). Soon transatlantic travel reversed as America's universities, research bodies, and public health institutions gained ground, establishing the lead paradigms and attracting students and scholars from all over the world.

Throughout the 20th century — a time of rich transatlantic exchange and rapid scientific progress — one thing remained constant: the very different understanding of the role of the state in the health of the public. Early on, Europe embarked on the road to the welfare state. Health is at the core of the European welfare state, albeit manifested in different models of organizing (and financing) the interface between public health and health care provision: the Scandinavian welfare state, the German health insurance model, and the British National Health Service. In contrast, the United States has long held the opposite understanding of the role of the state in health: that health is the responsibility of the individual, not of the state (2).

When considering the eight recommendations of the National Expert Panel on Community Health Promotion for the Centers for Disease Control and Prevention's National Center for Chronic Disease Prevention and Health Promotion (3), why raise history? Because public health debates are never strictly medical or scientific or based on evidence alone. They are subject to political culture, ideology, and heated argument. During the 1960s and 1970s, when the new epidemics of noncommunicable diseases were identified, the debate on disease causation raged between proponents of individual behavior modification and proponents of broader social interventions. The powerful U.S. institutions and large-scale intervention studies clearly stood for individual behavior modification while the European public health model — upheld in particular by the World Health Organization (WHO) — maintained that social conditions matter (4). One outcome of the exploration for a modern answer to social medicine was the Ottawa Charter for Health Promotion, adopted by an international WHO conference in Ottawa, Ontario, Canada, in November 1986 (5,6).

The Ottawa Charter states that health "is created and lived by people within the settings of their everyday life; where they learn, work, play and love" (6). The charter defines five major areas of action: healthy public policies, supportive environments, personal skills, community action, and reorientation of health services (5). It aims to combine a social determinants approach with a commitment to individual and community empowerment. Lester Breslow has argued that the Ottawa Charter is a seminal document and is the expression of a third public health revolution — one that perceives health as a resource (e.g., transportation, housing, education). Despite Breslow's endorsement, the charter never really caught on in the United States (7).

However, Europe — not the vague cultural and geopolitical notion but its concrete expression as the European Union (EU) — is moving forward in adopting the social paradigm outlined in the Ottawa Charter. Three recent developments illustrate how Europe is adopting the model.

One, the European Commission's Health and Consumer Protection Directorate General is in the process of drafting an overarching health strategy, which is scheduled to be adopted in June 2007 (8). The strategy follows a consultation process implemented throughout Europe and resulting in the publication of *Enabling Good Health for All — A Reflection Process for a New EU Health Strategy* by the former European Commissioner of Health and Consumer Protection (9). The strategy proposes a determinants-based approach to respond collectively to three major challenges: to reduce health gaps and improve the overall health of European citizens, to improve health through all sectors, and to achieve greater interaction by the EU at the global level.

Two, the recent Finnish presidency of the EU selected a determinants-based approach to health as its priority. This led to the adoption of a conclusion of the Council of the European Union, *Health in All Policies (HiAP)* (10). The conclusion reinforces Article 152 of the treaty that established the European Community, which states that "a high level of human health protection shall be ensured by all Community institutions in the definition of all Community policies and activities" (10). The council conclusion stresses that "many health determinants are to be found outside health care services" and that "everyday environment . . . ha[s] significant effects on health" (10).

Three, an exemplar for a national determinants-based health policy is the Swedish public health policy, Health on Equal Terms, which was adopted by the Swedish Parliament in 2003 (11). The overall objective of the policy is "to create social conditions that will ensure good health for the entire population" (11). The policy identified 11 domains of objectives and introduced new forms of public health reporting for mapping the contribution of other sectors to health, including "participation and influence in society" and "healthy and safe environments and products" (11).

These three developments are reflected in the emerging focus of European public health organizations. The theme for the European Public Health Association (EUPHA) conference held in Montreux, Switzerland, in November 2006 was "Politics, Policies and/or the Public's Health," and the EUPHA has issued *10 Statements on the Future of Public Health in Europe* (12). Perhaps the emerging focus represents the start of a new convergence and opportunity. "Politics, Policy and Public Health" is the theme of the annual meeting of the American Public Health Association in November 2007 in Washington, DC — what better venue to move the transatlantic dialogue forward and to engage in renewed learning from each other?

## References

[1] Barry JM (2004). The great influenza: the epic story of the deadliest plague in history.

[2] Esping-Anderson G (1990). The three worlds of welfare capitalism.

[3] Navarro A, Voetsch K, Liburd L, Bezold C, Rhea M (2006). Recommendations for future efforts in community health promotion: report of the National Expert Panel on Community Health Promotion.

[4] (1991). Health for all targets: the health policy for Europe.

[5] Kickbusch I (2003). The contribution of the World Health Organization to a new public health and health promotion. Am J Public Health.

[6] (1986). Ottawa charter for health promotion. Health Promot.

[7] Breslow L (1999). From disease prevention to health promotion. JAMA.

[8] (2005). Health in Europe: a strategic approach. Discussion document for a health strategy.

[9] Byrne D (2004). Enabling good health for all: a reflection process for a new EU health strategy.

[10] Council of the European Union. Council conclusions on Health in All Policies (HiAP). Proceeding of the 2,767th Employment, Social Policy, Health and Consumer Affairs Council meeting.

[11] Hogstedt C, Lundgren B, Moberg H, Pettersson B, Agren G (2004). The Swedish public health policy and the National Institute of Public Health. Scand J Public Health Suppl.

[12] (2005). 10 Statements on the future of public health in Europe: EUPHA reports 2004-1.

